# Novel Wireless-Communicating Textiles Made from Multi-Material and Minimally-Invasive Fibers

**DOI:** 10.3390/s141019260

**Published:** 2014-10-16

**Authors:** Stepan Gorgutsa, Victor Bélanger-Garnier, Bora Ung, Jeff Viens, Benoit Gosselin, Sophie LaRochelle, Younes Messaddeq

**Affiliations:** 1 Centre for Optics, Photonics and Lasers (COPL), Université Laval, Quebec, QC G1V 0A6, Canada; E-Mails: sgorgutsa@gmail.com (S.G.); victor.belanger-garnier.1@ulaval.ca (V.B.-G.); jfviens@copl.ulaval.ca (J.V.); Sophie.Larochelle@gel.ulaval.ca (S.L.); 2 Department of Electrical and Computer Engineering, Université Laval, Quebec, QC G1V 0A6, Canada; E-Mail: Benoit.Gosselin@gel.ulaval.ca; 3 Department of Physics, Engineering Physics and Optics, Université Laval, Quebec, QC G1V 0A6, Canada; 4 Department of Electrical Engineering, École de Technologie Supérieure, Montreal, QC H3C 1K3, Canada; E-Mail: Bora.Ung@etsmtl.ca

**Keywords:** wearable antennas, multi-material fibers, bio-textiles

## Abstract

The ability to integrate multiple materials into miniaturized fiber structures enables the realization of novel biomedical textile devices with higher-level functionalities and minimally-invasive attributes. In this work, we present novel textile fabrics integrating unobtrusive multi-material fibers that communicate through 2.4 GHz wireless networks with excellent signal quality. The conductor elements of the textiles are embedded within the fibers themselves, providing electrical and chemical shielding against the environment, while preserving the mechanical and cosmetic properties of the garments. These multi-material fibers combine insulating and conducting materials into a well-defined geometry, and represent a cost-effective and minimally-invasive approach to sensor fabrics and bio-sensing textiles connected in real time to mobile communications infrastructures, suitable for a variety of health and life science applications.

## 1. Introduction

As the relationship between humans, computers, and machines grows towards an uninterrupted and ever-closer level, mobile biomedical devices such as bio-sensing textiles will prompt many new ubiquitous mobile applications on a global scale such as 24-h medical monitoring, medical emergency communications, and patient data recording, to name a few. In fact, clothing and other wearable garments are increasingly set to become platforms for arrays of biomedical sensors, transducers and microprocessors that may continuously monitor health while interacting cost-effectively with the user, service provider, and the cloud. Such garments are often called *smart textiles*.

### 1.1. Smart Textiles in Biomedical Applications

Smart textiles are based on research occurring in different disciplines: chemistry, physics, material science, and computer science and technology. Significant for this research is the interdisciplinary approach and the interaction between basic research and design activities. Textiles of today are materials with applications in almost all our activities, we wear clothes all the time and we are surrounded with textiles in almost all our environments. The integration of multifunctional use in such a common material has become a special area of interest in recent years. Fiber yarns, and fabrics [1] with added functionality have been developed for a wide range of applications in sport training [2,3] and healthcare industries [4], fashion [5], work ware and military [6,7]. It has been shown that various optical and electrical functionalities may be delivered at length scales and in a mechanically flexible form associated with fibers [8–10]. Besides sensing, reacting, and conducting electricity, the next-generation textile will be able to perform computational operations [10], thus shifting textiles from passive functionalities to a more dynamical role. Active functionalities in a smart textile may include power generation or storage, human interface elements, bio-sensing devices, radio frequency (RF) emission/reception, various assistive technologies such as personal emergency awareness systems and response communication. This new generation of textiles will favor the interaction between users and their environment by combining novel materials and integrated computing power, and should find numerous applications in almost every aspect of our lives.

**Figure 1.**
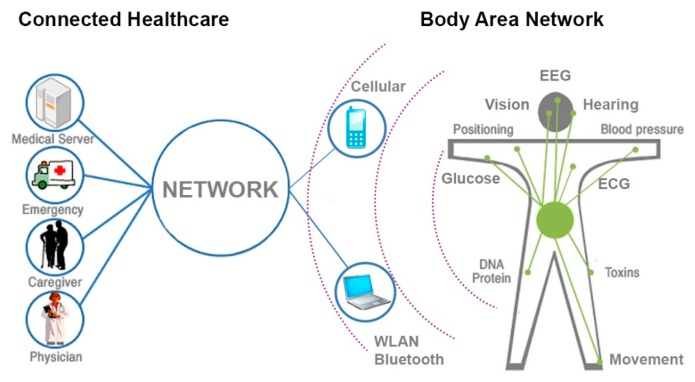
Multi-material fibers weaved into textiles are suitable for a variety of health and life science applications pertaining to bio-sensing textiles connected through wireless networks.

Textile-based sensing has always been a large field of research in the biomedical community and has constantly attracted considerable attention. Up to date it was demonstrated that capacitive switches can be used for electrocardiogram (ECG) [11] and electromyography (EMG)—measuring activity produced by skeletal muscles [12], and electroencephalography (EEG) sensing [13]; fabrics incorporating thermocouples can be used for sensing temperature [14]; luminescent elements integrated in fabrics could be used for bio-photonic sensing [15]; shape-sensitive fabrics can sense movement [16], and can be combined with EMG sensing to calculate muscle fitness, *etc.* Thus, there is a great variety of textile bio-sensors that could be integrated into textiles, suitable for a variety of ubiquitous mobile applications pertaining, for example, to 24/7-connected healthcare and body area networks, which require delivery of wireless RF signals, as schematized in Figure 1. These applications in connected healthcare rely on WLAN, cellular networks, or other wireless communication bands to be effective 24 h per day, and thus require RF antenna integration into textiles.

### 1.2. Textile Antennas and Multi-Material Fiber Approach

In the past few years, several prototypes of RF textiles have been demonstrated, still the current trend of wireless textile communications devices is geared towards obtrusive monolithic patch designs. The most common approach relies on regular conductive threads [17] or coated polymer fibers [18] sewed in a shape of a patch antenna on top of the textile. There were also several attempts to integrate microstrip antennas or patch-like sensors not just between the layers or on top of a textile, but also literally inside the textile via 3D weaving [19] technique. Recently, Whittow *et al.* [20] demonstrated a textile patch antenna produced by inkjet-printing technique, though the authors admit that such antennas are prone to damage, operation frequency shifts and losses due to compressing and tensioning of the conducting surface.

Investments in both commercial and academic research have been driven mainly by leveraging the CMOS approach and further miniaturization of the electronics to obtain smart textile functionalities. In the CMOS approach, planar electronics and optoelectronic devices based on conventional microelectronics manufacturing processes are inserted in a hybrid manner into the threads of the textile. With this approach, practical issues have hindered the wide adoption of smart textile technology, such as:

*Problem of manufacturability:* The process for mass producing traditional textiles does not translate well to smart textiles containing CMOS components—cutting patterns from larges rolls of cloth and sewing them together to make garments breaks and reforms electronic connections in an uncontrolled manner. Generally, since smart fabric elements have to be introduced onto a textile surface in a separate post-processing step, this makes fabrication of RF textiles labor intensive and expensive.

*Problem of environmental endurance:* Clothing and some other textile products must be washable, which subjects the electronics elements to water and chemical immersion, physical stress, and extreme temperatures—the current state of the art tends to be too fragile for this treatment, especially for smart textiles containing CMOS components. The state of the art in flexible electronics and nanotechnology, required to integrate power and computer processing elements into fabric, has not advanced to the point that analog processing or digital logic can be integrated into fabrics.

Multi-material fibers have the potential to address some of the major challenges in developing sensor fabrics and bio-sensing textiles connected in real time to mobile communications infrastructures. By combining insulating and conducting materials into a well-defined geometry imbedded in a fiber, many functional electronic devices are achieved, in particular fiber and textile devices that lend themselves to RF-emission adaptable to existing broadband mobile infrastructures. Research in co-drawn metal-insulator-semiconductor photo-detecting fiber devices with mesoscopic-scale cross-sectional features has heralded a novel path to acoustic, thermal, and optical detection [8–10]. In such fibers, various functionalities may be delivered at length scales and in a mechanically flexible form. Thus, multi-material fibers provide a perfect building material for the next generation of biomedical textiles as they can be easily integrated during the weaving process in a cost-effective and minimally-invasive manner while preserving the mechanical and cosmetic properties of the garments.

## 2. Fiber-Based RF-Antennas and Interconnects

In this work, we propose to implement the core functionalities, *i.e.*, signal delivery and radiative emission at 2.4 GHz frequency, and electrical interconnects, within the sub-millimeter fibers themselves which are weaved into the threads of a fabric.

### 2.1. Leaky Coaxial Cable Antenna

Leaky Coaxial Cables (LCXs) are known for their capability of distributing radio waves in tunnels, mines, underpasses, and many other confined environments, with smooth electrical field coverage [21]. There are very few studies on the use of small-scale LCXs for novel mobile communications systems such as biomedical textiles. Fiber RF-antennas based on LCXs can be achieved through combinations of materials and careful design to impart fiber RF-functionality, while preserving fiber sub-millimeter size and flexibility suitable for integration into textiles.

**Figure 2.**
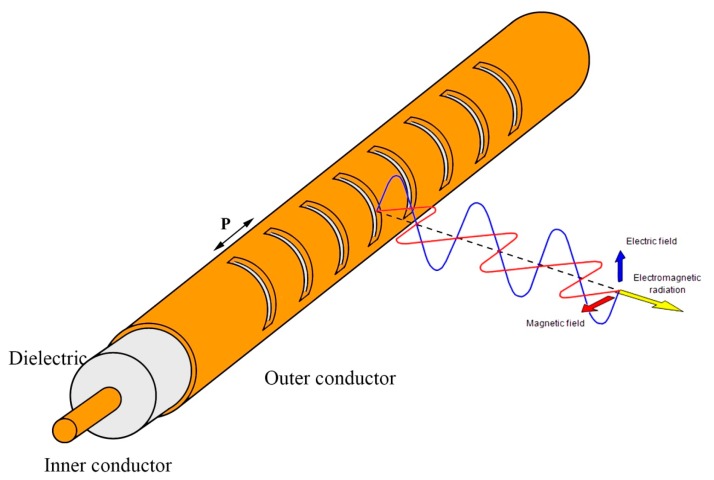
Schematic of the leaky coaxial antenna (LCX).

LCXs generally consist of three parts: an inner conductor, a dielectric material, and an outer conductor that exhibits slits at a prescribed periodic distance *P* [21–24], as schematized in Figure 2. The periodic distance *P* and the relative permittivity ε*_r_* of the dielectric must be designed in accordance with the frequency constraints of the wireless networks as well as with the clothing material and dimensional constraints of the garment industry. Usually, the theoretical modeling considers an infinitely long coaxial cable that supports a single TEM propagation mode. This allows the use of Bloch-Floquet theory, which greatly eases the analysis of the radiation properties. The geometric parameters of the LCX are generally chosen as to allow only the *m =* −1 fundamental spatial harmonic [21], which radiates at an angle of:

$$\phi_{m}={\sin^{-1}\left(\sqrt{{ɛ}_{r}}+m\lambda /P\right)|}_{m=-1}=\sin^{-1}\left(\sqrt{{ɛ}_{r}}+\lambda /P\right)$$

This mode of operation provides a nearly transverse (*i.e.*, broadside) radiation pattern. The frequency range of LCXs is determined by the periodic distance *P* of the slits and the relative permittivity *ε_r_* of the dielectric, defined for the *m* = −1 fundamental spatial harmonic by the inequality:

$$\frac{c}{(\sqrt{{ɛ}_{r}+1})P}\leq f\leq \frac{c}{(\sqrt{{ɛ}_{r}+1})P}$$

The fiber antenna itself may represent only a few small threads in the textile fabric, thus enabling the use of non-standard textile fiber materials, such as polymer-clad silica fibers, without disrupting the mechanical and cosmetic attributes of the garment. This opens the possibility of leveraging the polymer-clad silica fiber manufacturing capabilities of the fiber optics industry. Such fibers (*ε_r_* ∼ 3.7) provide a periodic distance *P* in the range from 1 to 40 cm, for carrier frequencies in the range of 0.8–5.0 GHz, respectively, which is compatible with typical clothing dimensions. Therefore, the LCXs can be designed for textile antenna applications with the WLAN 802.11b/g/n 2.4 GHz ISM band in mind, and can be scaled according to any desired carrier frequency of most wireless networks while leveraging the economies of scale of polymer-clad silica fiber industry. A transmission loss of a few dB/m for the coaxial cable is acceptable for textile antenna applications where typical clothing dimensions are below the meter range.

## 2.2. Dipole Fiber Antenna

Dipole antenna is probably one of the simplest and most widely used types of antennas, which is ideally suited for fiber antennas. Henceforth, by the term *dipole*, we will mean a center-fed half-wave dipole. Naturally, a dipole antenna that consists of a pair of tubular conductors of a certain diameter and length (l = *λ*/2) aligned in tandem so that there is a small feeding gap at the center can be fabricated using the same polymer-clad silica fibers as for LCXs.

## 3. Fabrication

**Figure 3.**
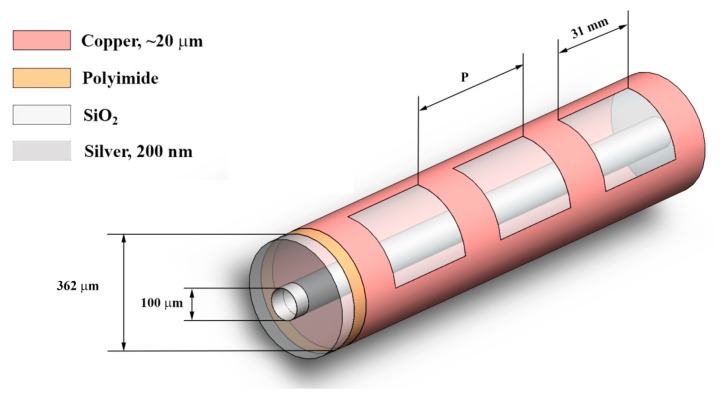
Schematic and radii of the fabricated 3-slit LCXs using hollow-core polymer-clad silica fibers and operating at 2.4 GHz. A 20 μm layer of protective acrylic coating was deposited over the fiber except for two 2 mm endpoints to allow for electrical connections.

LCXs were fabricated using a hollow-core silica fiber with an inner radius of 100 μm and outer radius of 181 μm, coated with an 18 μm thick polyimide layer, and where the inner and outer surfaces of the hollow-core polyimide-silica fiber were coated with thin films of silver and copper metal, respectively, as schematized in Figure 3.

**Figure 4.**
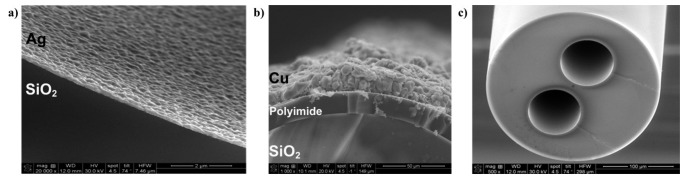
SEM pictures of the (**a**) LCX inner silver thin film lining; (**b**) LCX outer copper thin film lining; (**c**) 75µm-diameter dual-core fiber interconnect.

**Figure 5.**
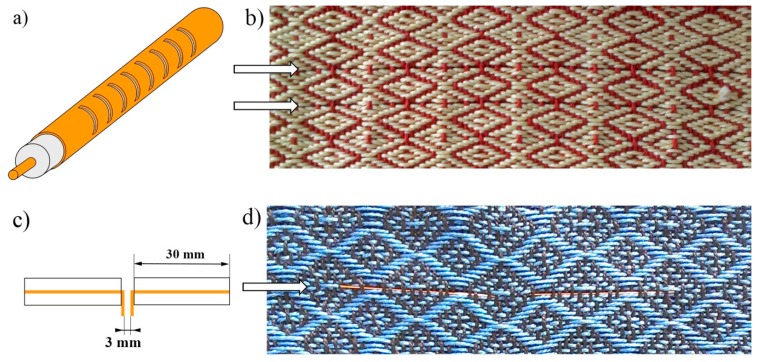
LCX (**a**) and dipole; (**b**) and cotton; (**c**) antennas (arrowed) weaved into the threads of a wool; (**d**) textile fabric. Both antennas were fabricated using polymer coated hollow-core silica glass fibers with an inner radius of 100 μm and outer radius of 181 μm.

Silver metal coating was obtained using Tollen's reaction [25], a redox chemical reaction that consists in the precipitation of an aqueous solution composed of a mixture of silver nitrate (AgNO_3_), potassium hydroxide (KOH), dextrose (C_6_H_12_O_6_), and ammonium hydroxide (NH_4_OH). This resulted in the oxidation of the aldehyde to a carboxylic acid, and the subsequent reduction of the complex to silver metal: 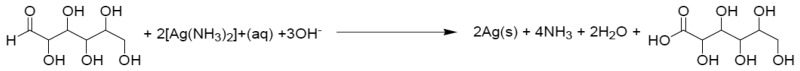


The aqueous solution was injected into the inner hollow capillary at 500 μL/min rate using a motorized precision syringe, providing a flexible inner silver thin film lining of about 0.2 μm thickness with good adhesion to silica glass, as shown in Figure 4. The outer conductor of the LCX consisted of a 20 μm thick electro-deposited copper metal thin film, as shown in Figure 5. Electro-deposition was performed in an acid copper bath containing a solution of 0.56 M of CuSO_4_ · 5H_2_O maintained at pH = 3 using concentrated H_2_SO_4_. The resulting copper and silver linings displayed good adhesion to their respective substrates. Electrical DC resistances of 3.0 Ω · cm^−1^ and 95.4 mΩ · cm^−1^ were measured for the inner silver and outer copper layers, respectively, providing good electrical matching to the standard 50 Ω impedance of electronics components. Finally, a thin layer of protective acrylic coating was deposited over the entire 250 mm-long LCX fiber except for two 2 mm endpoints to allow for SMA electrical connections. Since the inner core of the LCX fiber remained hollow, the endpoint male electrical connection was formed by a tin-coated copper wire (ø 127 μm) inserted through the inner hollow core. In the present configurations, all electrical connections were made manually by direct soldering.

Dipole fiber antennas were fabricated using the same hollow-core silica fibers and according to the same procedure for the silver deposition of the inner electrode, and since there is no need for external electrode for the dipole antenna, the polyimide protective coating was left intact.

The conductor elements of the fiber antennas are embedded within the fibers themselves, providing electrical and chemical shielding against the environment. The electrical conductivity of silver and copper metal coatings remained stable during standard heat and humidity tests, which consisted of exposing the fibers to +60 °C temperature and 80% relative humidity for 96 h. These coatings provided suitable conductor elements for long electrical interconnects imbedded within the fiber. Multi transmission lines can be fabricated using hollow-core fibers such as the dual-core interconnect depicted in Figure 4c. The two 75 μm-diameter hollow cores were coated with a silver lining providing a DC electrical resistance of about 100 Ω over a length of 1 m, which is suitable for a variety of applications pertaining to textile bio-sensor interconnects.

Hollow-core polymer-clad silica fibers can withstand high tensile and bending stresses (GPa), high temperature operation (350 °C), mechanical abrasion and harsh environmental exposure (heat-humidity tests, water, detergent, acids) compatible to most textiles applications, due to the thick polymer (e.g., polyimide) overcoat that reinforces significantly fiber glass brittleness. Of concern is the consideration of minimum bending radius (in this case R ≥ 1 cm); the fiber can be broken on purpose over a sharp edge but it will withstand stepping upon by a person. In general, the approach of using silica glass fibers was previously justified [26] where it was demonstrated that polymer and glass optical fibers can be successfully integrated into textiles, garment and even paper to act as displays, and that mechanical properties of such fibers allow them to be used in the industry grade weaving looms.

## 4. Textile Integration

A computerized loom from AVL Looms Inc., featuring a 40 cm weaving width and eight harnesses, was used to integrate the LCX and dipole fiber antennas into a textile fabric. This allowed the design and production of smart RF textiles while leveraging a method of mass producing traditional textiles. The fiber antennas themselves represent only a few small substitution threads in the textile fabric, as shown in Figure 5, and appeared to be flexible and unobtrusive enough to be essentially indistinguishable from the textile host, thus providing a minimally-invasive attribute to the textile integration.

Two different textile prototypes were fabricated for LCX (Figure 5b) and dipole (Figure 5d) fiber antennas, in first case relatively thick (12 end-per-inch or EPI) wool threads were used, while the second textile sample was weaved using thin (EPI = 22) cotton threads. In both cases, the LCX and dipole fibers withstood conventional weaving process and textile manipulation without any damage or performance losses.

Since fibers are inherently congenial to textile manufacturing, the process for mass producing traditional textiles is not disrupted significantly by the use of sub-millimeter-size multi-material fibers, which can have the same mechanical flexibility as traditional textile threads. This represents a cost-effective and minimally-invasive approach to fabricating transmitters for bio-sensing textiles. Two significant cost benefits can be obtained from multi-material fibers: (1) these fibers are congenial to the textile industry and can be adapted directly to traditional industrial looms; and (2) they benefit from the economies of scale of the fiber optics manufacturing industry. In addition, by using chemically stable, mechanically strong, and thermally robust fiber cladding materials (such as silica glass and high-Tg polymers of Figure 3), the imbedded elements within the fibers can be shielded from water, detergent, and chemical exposure, physical stress, and extreme temperatures, while preserving the cosmetic appeal and mechanical properties of traditional textile threads.

## 5. Results and Discussion

### 5.1. LCX Fiber Antenna

**Figure 6.**
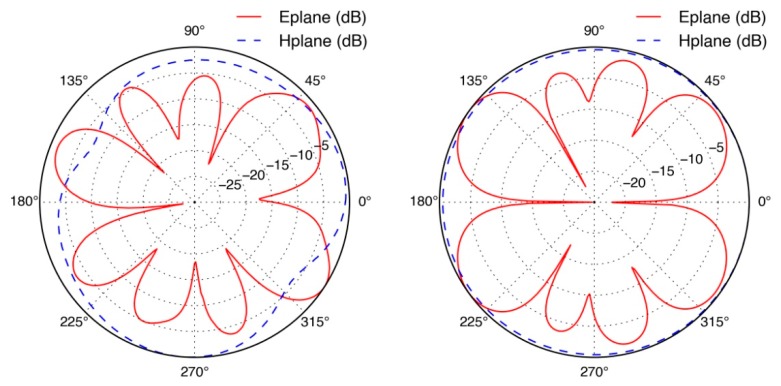
Measured (**Left**) and simulated (**Right**) radiation patterns along the E-plane and H-plane polarizations of the LCX textile fiber operating at 2.4 GHz frequency.

The radiation pattern measurements of the LCXs were obtained in an anechoic chamber using a wide-band Log periodic directional antenna Aaronia HyperLOG-7060 from 700 MHz to 6 GHz along with a tunable signal generator on the transmission side, while the LCXs acted as the far-field receiver. The LCX fibers were carefully positioned with the slits facing the transmitting antenna at 0° position. Radiation pattern measurements along the E-plane (parallel) and H-plane (perpendicular) polarizations are presented in Figure 6. In agreement with theoretical predictions, the LCXs exhibited an omnidirectional radiation pattern in the H-plane, while the E-plane presented several radiation lobes due to spatial harmonics of the LCX structure. Peak directivities in the E-plane were calculated at 6.8 dBi for the LCX operating at 2.4 GHz. Slight asymmetries in the radiation pattern of the LCX fiber may be attributed to roughness, thickness, and electrical conduction non-uniformities in the silver thin film lining of the LCXs. The sub-millimeter attribute of the coaxial design, the thinness of the conducting layers, and the inherent process variations of Tollen's silver nitrate reduction, render the LCX device sensitive to skin depth effects.

**Figure 7.**
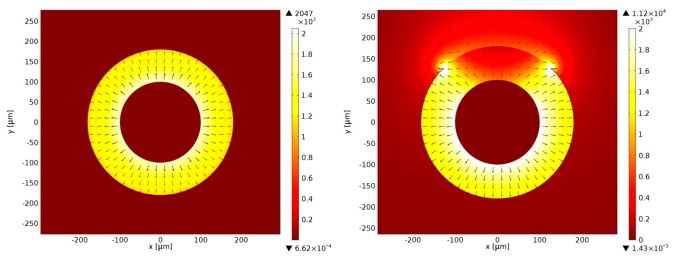
E-field simulation of the propagation of a TEM-like mode inside the windowless segments of the LCX (**Left**); and coupling to radiation modes in the windowed segments (**Right**).

Two-conductor transmission lines, such as LCXs, have a dominant quasi-TEM mode of propagation. The quasi-TEM assumption is generally valid for structures made with low-loss dielectrics and near-perfect conductors [27,28]. Due to the thinness of the LCX silver thin film lining, as shown in Figure 4b, which is significantly thinner than the skin depth of silver at 2.4 GHz frequency, the condition of near-perfect conductor is not strictly enforced. Finite element calculations were carried out using COMSOL Multiphysics software to verify that a TEM mode could still propagate despite the thinness of the silver layer. Effective mode indices of *n* = 1.94 − 0.17*j* and *n* = 1.90 − 0.16*j* were found for the window-less and the windowed portions of the LCX, respectively. The simulated electric field structure and intensities shown in Figure 7 displaying quasi-TEM mode attributes and the indices nearing the expected value of *n* = 1.94 are strong indicators of quasi-TEM mode propagation. The results confirm that a TEM-like mode propagates inside the windowless segments of the LCX, whereas coupling to radiation takes place in the windowed segments.

### 5.2. Dipole Fiber Antenna

**Figure 8.**
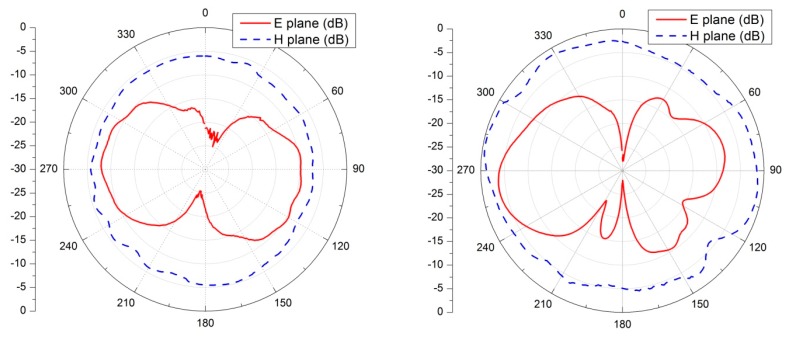
(**Left**) Radiation pattern of the commercial dipole WiFi antenna and (**Right**) radiation pattern of the textile integrated fiber dipole antenna, both operating at 2.45 GHz frequency.

Radiation patterns of the textile-integrated dipole fiber antennas were also measured in the anechoic chamber at 2.45 GHz frequency, using the technique outlined in Section 5.1. We have also directly compared their performance with the commercial dipole WiFi duck antennas. These antennas are widely used in multiple wireless consumer devices (WiFi access point, routers, *etc.*) and for communication systems as they are cheap, provide omnidirectional radiation pattern. Results of these measurements are shown in Figure 8, both commercial (Figure 8 left) and fiber (Figure 8 right) dipoles demonstrate classical *λ*/2 dipole radiation patterns.

### 5.3. Gain Measurements

**Figure 9.**
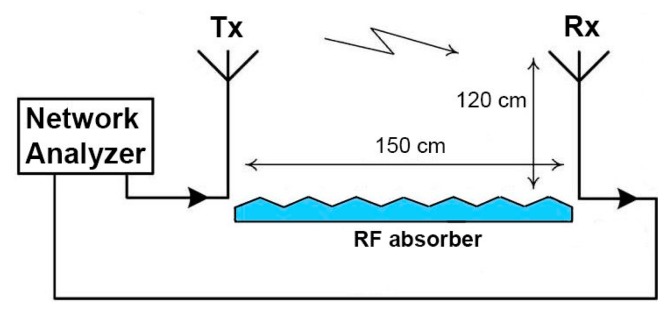
Schematic of the antenna gain measurements setup. Two antennas are mounted on bases, oriented in the direction of the maximum transmitted signal and connected to a network analyzer, and then are used for line-of-sight transmission in an unobstructed lab environment. RF absorber is placed on the ground in order to avoid multipath propagation.

Efficiency of an antenna (*η*) is related to its gain (*G*) and directivity (*D*) as G = *ηD* and relates the power delivered to the antenna to the power radiated or dissipated within the antenna *η = P_radiated_*/*P_input_*. In order to measure antenna gain experimentally the following technique, based on the well-known Friis equation, was used. Two antennas were mounted on bases, oriented in the direction of the maximum transmitted signal, connected to an Agilent 8722ES network analyzer, and used for line-of-sight transmission in an unobstructed lab environment over a distance of 150 cm, greater than the far field criterion at *f* = 2.45 GHz, and a height of 120 cm off the ground. Additionally, an RF absorber was placed on the ground in order to exclude any multipath signals, schematic of the setup is shown in Figure 9.

In this case, the ratio of power available at the input of the receiving antenna, *P_R_*, to output power of the transmitting antenna, *P_T_*, is given by the Friis equation:

$$\frac{P_{R}}{P_{T}}=G_{T}G_{R}{\left(\frac{\lambda }{4\pi R}\right)}^{2}$$

where *G_T_* and *G_R_* corresponds to the gain of the transmitting and receiving antennas. The Friis equation can be written also in terms of the scattering (S)-parameters as:

$${\left|S_{21}\right|}^{2}=\frac{P_{R}}{P_{T}}=\left(1-{\left|S_{11}\right|}^{2}\right)G_{T}G_{R}{\left(\frac{c}{4\pi Rf}\right)}^{2}$$

**Figure 10.**
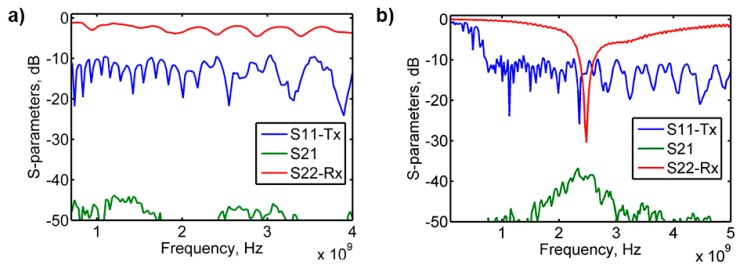
Typical results of the gain measurements for the LCX (**a**) and dipole (**b**) textile integrated fiber antennas. The S11 curves (blue) correspond to the return losses of the Aaronia HyperLog 7060 antenna and S_22_ curve (red) to the return losses of the antenna under study, the green S_21_ curve represents the power transmitted from one antenna to another over the 150 cm distance in unobstructed lab environment. Antenna gains were measured to be 1.62 dBi for the LCX antenna, and at 3.34 dBi for the dipole antenna.

Thus, it becomes possible to determine the gain of the antenna-under-test either by using a well-characterized transmitting antenna with known gain or by using to identical unknown antennas. In our case, we again used the Aaronia HyperLog 7060 antenna as a transmitter and dipole antennas acting as unknown receivers. Measured S-parameter curves for the textile-integrated LCX and dipole fiber antennas are shown in Figure 10; S_11_ curve (blue) corresponds to the return losses of the Aaronia HyperLog 7060 and S_22_ curve (red) to the return losses of the textile-integrated fiber antenna, the green S_21_ curve represents the power transmitted from one antenna to another.

Using the described technique, we have calculated the gain of the textile-integrated fiber dipole as 3.34 dBi and the gain of the commercial duck antenna as 3.45 dBi, which is close to the 3.5 dBi value provided by the manufacturer. Gain of the LCX fiber antenna was estimated as 1.62 dBi, which, although lower than typical values for the antennas in mobile applications, is still sufficient to act as a receiver.

These antennas have omnidirectional radiation patterns; therefore part of the radio waves emitted by the antennas would be absorbed by the human body. The rate at which energy is absorbed by the human body is measured by the Specific Absorption Rate (SAR), and its maximum levels for modern wearable devices have been set by governmental regulating agencies in many countries. In the USA, the Federal Communications Commission (FCC) has set a SAR limit of 1.6 W/kg, averaged over a volume of 1 g of tissue, for the head. In Europe, the limit is 2 W/kg, averaged over a volume of 10 g of tissue. Antenna simulations performed with the ANSYS HFSS software show that, with the current dipole configuration, the SAR is less than 1.6 W/kg when radiated power is −8 dBm at 2 mm distance from the skin surface of a human body. This would allow the RF-textile to maintain good-to-excellent signal strength (>−60 dBm) on the receiver part for most commercial WIFI router antennas.

## 6. Conclusions

In this work, we have demonstrated novel textile fabrics integrating unobtrusive multi-material fibers that communicate through 2.4 GHz wireless networks with excellent signal quality. The conductor elements of the textiles are embedded within the fibers themselves, providing electrical and chemical shielding against the environment, as well as electrical impedance matching to standard RF devices and systems, while preserving the mechanical and cosmetic properties of the garments. These multi-material fibers combine insulating and conducting materials into a well-defined geometry, and represent a cost-effective and minimally-invasive approach to sensor fabrics and bio-sensing textiles connected in real time to mobile communications infrastructures, suitable for a variety of health and life science applications.

## References

[1] Cork C.R., Dias T., Acti T., Ratnayaka A., Anastasopoulos I.E.M., Piper A. The next generation of electronic textiles.

[2] Tang S.L.P., Stylios G.K. (2006). An overview of smart technologies for clothing design and engineering. Int. J. Cloth. Sci. Technol..

[3] Morris D., Schazmann B., Wu Y., Coyle S., Brady S., Hayes J., Slater C., Fay C., Lau K.T., Wallace G. Wearable Sensors for Monitoring Sports Performance and Training.

[4] Paradiso R., Loriga G., Taccini N. (2005). A wearable health care system based on knitted integrated sensors. IEEE Trans. Inf. Technol. Biomed..

[5] Berzowska J. Very slowly animating textiles: Shimmering flower.

[6] Dumas J., Defence R., Canada D. (2009). Textile J..

[7] Dumas J. The Challernges of Modern Military Ground Operations in Arctic Regions Expo Hightex and Advanced Workwear Canada CTT Group Montreal, QC, Canada 18–20 3 2014

[8] Bayindir M., Sorin F., Abouraddy A.F., Viens J., Hart S.D., Joannopoulos J.D., Fink Y. (2004). Metal-insulator-semiconductor optoelectronic fibres. Nature.

[9] Bayindir M., Abouraddy A.F., Shapira O., Viens J., Saygin-Hinczewski D., Sorin F., Arnold J., Joannopoulos J.D., Fink Y. (2006). Kilometer-Long Ordered Nanophotonic Devices by Preform-to-Fiber Fabrication. IEEE J. Sel. Top. Quantum Electron..

[10] Sorin F., Abouraddy A.F., Orf N., Shapira O., Viens J., Arnold J., Joannopoulos J.D., Fink Y. (2007). Multimaterial Photodetecting Fibers: a Geometric and Structural Study. Adv. Mater..

[11] Cheng J., Amft O., Lukowicz P. Active capacitive sensing: Exploring a new wearable sensing modality for activity recognition.

[12] Linz T., Gourmelon L., Langereis G. Contactless EMG sensors embroidered onto textile.

[13] Chi Y.M., Cauwenberghs G. Wireless Non-contact EEG/ECG Electrodes for Body Sensor Networks.

[14] Kinkeldei T., Zysset C., Cherenack K., Tröster G. A textile integrated sensor system for monitoring humidity and temperature.

[15] Omenetto F., Kaplan D., Amsden J., Negro L.D. (2013). Silk Based Biophotonic Sensors.

[16] Hu J. (2007). Shape Memory Polymers and Textiles.

[17] Osman M.A.R., Rahim M.K.A., Samsuri N.A., Ali M.E. Compact and embroidered textile wearable antenna.

[18] Lanlin Z., Zheyu W., Volakis J.L. (2012). Textile Antennas and Sensors for Body-Worn Applications. IEEE Antennas Wirel. Propag Lett..

[19] Yao L., Jiang M., Zhou D., Xu F., Zhao D., Zhang W., Zhou N., Jiang Q., Qiu Y. (2011). Fabrication and characterization of microstrip array antennas integrated in the three dimensional orthogonal woven composite. Compos Part B Eng..

[20] Whittow W.G., Chauraya A., Vardaxoglou J.C., Yi L., Torah R., Kai Y., Beeby S., Tudor J. (2014). Inkjet-Printed Microstrip Patch Antennas Realized on Textile for Wearable Applications. IEEE Antennas Wirel. Propag. Lett..

[21] Hong W.J., Mei K.K. (2001). Theory and analysis of leaky coaxial cables with periodic slots. IEEE Trans. Antennas Propag..

[22] Delogne P.P., Laloux A.A. (1980). Theory of the Slotted Coaxial Cable. IEEE Trans. Microw. Theory Tech..

[23] Kim D.H., Eom H.J. (2007). Radiation of a Leaky Coaxial Cable with Narrow Transverse Slots. IEEE Trans. Antennas Propag..

[24] Addamo G., Orta R., Tascone R. (2008). Bloch Wave Analysis of Long Leaky Coaxial Cables. IEEE Trans. Antennas Propag..

[25] Benet W.E. (2011). The mechanism of the reaction of the Tollen reagent. J. Chem. Res..

[26] Gorgutsa S., Berzowksa J., Skorobogatiy M., Kirstein T. (2013). 3-Optical fibers for smart photonic textiles. Multidisciplinary Know-How for Smart-Textiles Developers.

[27] Clayton P.R. (2008). Analysis of Multiconductor Transmission Lines.

[28] Cheng D.K. (1999). Field and Wave Electromagnetics.

